# Environmental Factors as a Cause of Differences in the Feet of Ecuadorian Children and Its Relation to Their Footwear

**DOI:** 10.3390/children8060459

**Published:** 2021-05-31

**Authors:** Laura Martin-Casado, Christian Barquín, Alberto Aldana-Caballero, Felix Marcos-Tejedor, Xavier Aguado

**Affiliations:** 1 Department of Nursing, Physiotherapy and Occupational Therapy, Faculty of Health Sciences, University of Castilla-La Mancha, Talavera de la Reina, 45600 Toledo, Spain; laura.martincasado@uclm.es (L.M.-C.); Alberto.Aldana@uclm.es (A.A.-C.); 2 Department of Sport Science, Faculty of Education, Technical University of Ambato, 180202 Ambato, Ecuador; c.bzr10@hotmail.com; 3 Department of Medical Sciences, Faculty of Health Sciences, University of Castilla-La Mancha, Talavera de la Reina, 45600 Toledo, Spain; 4 Department of Physical Activity and Sport Science, Faculty of Sports Sciences, University of Castilla-La Mancha, 45001 Toledo, Spain; xavier.aguado@uclm.es

**Keywords:** climatic variety, children, footwear, foot dimensions, 3D foot digitizer

## Abstract

The objective of this study was to analyze the differences in foot measurements of Ecuadorian children according to their geographical location of residence, taking into account climatic differences (1). A total of 1662 children (2) participated in the study. Three groups were established: coast, mountains and amazonia. The type of footwear (3) used was recorded and the lengths, widths, perimeters, heights and angles of both (4) feet were analyzed with a 3D foot digitizer (5). The variable ‘fitting of the foot to footwear’ was also obtained. Children living in coastal areas presented greater lengths, widths, perimeters and heights compared to those living in the mountain (*p* > 0.001) and amazonia (*p* < 0.001) between ages 5 to 13. Mountain residents showed a greater first toe angle than coast residents (*p* > 0.001) aged 8 to 17. Children used shoes smaller than required by their foot length (*p* < 0.01). Ecuadorian children from the coast presented longer and wider feet with higher foot arches, whilst those from mountains presented greater first toe angle. The studied sample used footwear up to one size smaller than the size corresponding to their foot length. The fitting and type of footwear used according to climatic differences could be interfering with normal foot development.

## 1. Introduction

The physiological development of the lower limbs in children, and especially the foot, involves a sequence of consecutive morphological changes which can be compromised by external factors such as footwear. This development can be also altered by social-environmental and demographic factors [1,2,3,4,5]. Previous studies concluded that school-aged German children present flatter and longer feet [6] and morphological differences depending on their ethnic background [7]. On the other hand, Sacco et al. [8] described that Brazilian children aged 5 to 10 presented narrower forefeet than German children. Other authors analyzed differences in foot dimensions of children living in urban and rural areas, concluding that children living in urban areas presented a higher rate of flat feet [3,9,10].

Despite the spread in the last few years of 3D scanning to analyze the morphological dimensions of the foot [1,11,12,13], the results of most studies are based on the analysis of the children’s footprint [3,8,9,10], which does not yield three-dimensional data and therefore makes it impossible to obtain volumetric measurements to correlate to footwear. Flexibility and function of the shoes that allow normal foot movement, especially around the forefoot, is of paramount importance for the health of the foot in children [14]

It is worth pointing out that no studies were found on the morphology of children’s feet in the Ecuadorian population, which has important physical, social and geographical differences. Ecuador is a multiethnic country, whose peninsula is divided into three main natural regions which have large cultural and climatic differences due to the altitudes where each of them is located: coast (between 0 to 300 m), mountains (between 800 to 6500 m) and Amazonia (between 100 to 800 m).

Based on that, the aim of this study was to analyze the feet of school-aged Ecuadorian children, both morphologically and anthropometrically, according to their age and geographical area, by using 3D foot scanning.

## 2. Materials and Methods

### 2.1. Sample

**Figure 1:**
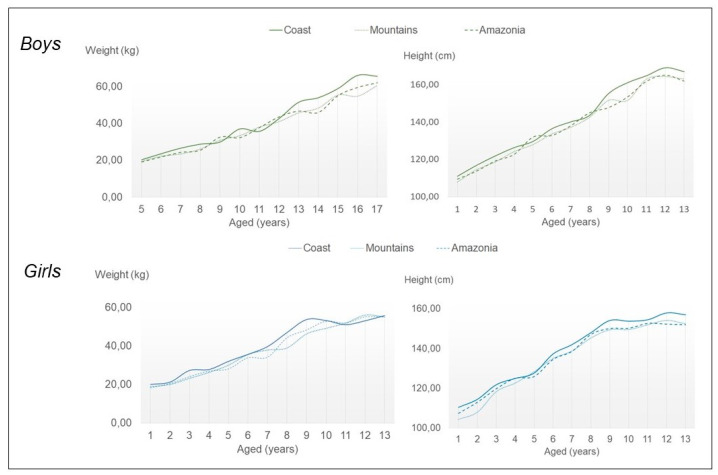
Height and weight boys and girls in coast, mountains and amazonia.

A total of 1662 boys and girls aged between 5 and 17 from different education units in Ecuador were analyzed (Figure 1). Three groups were created based on the area of residence: coast (boys: *n* = 258; girls: *n* = 291), mountains (boys: *n* = 314; girls: *n* = 272) and Amazonia (boys: *n* = 246; girls: *n* = 281). The exclusion criteria were: neurological alterations, musculoskeletal disorders, or other conditions affecting their feet.

The study was approved by the Bioethics Committee of the University of San Francisco de Quito (2016-083E). The children’s legal guardians signed an informed consent form and the Declaration of Helsinki was complied with at all times.

### 2.2. Measurements

The children’s heights and weights were measured using a measuring rod, model 420KLWA (WelchAllyn, Chicago, IL, USA). Foot dimensions were obtained with the 3D digitizer model IFU-S-01 (INFOOT, Osaka, Japan), following the protocol below [15]:

Pre-scanning the foot, 13 skin labels were placed to absorb the light from the scanner, following the manufacturer’s instructions, in: pternion, sphyrion fibulare, navicular, landing points, sphyrion, medial point of heel breadth, the most medial point of medial malleolus, the most lateral point of lateral malleolus, tuberosity of the fifth metatarsal, metatarsale tibiale, metatarsale fibulare, first toe joint, fifth toe joint; whit the subject seated, barefoot, in neutral position on the ground. Later, with the subject in a standing position, with both hands on a handrail and their gaze fixed on a target placed 0.5m away at eye level, both feet were consecutively scanned.

### 2.3. Variables

**Figure 2:**
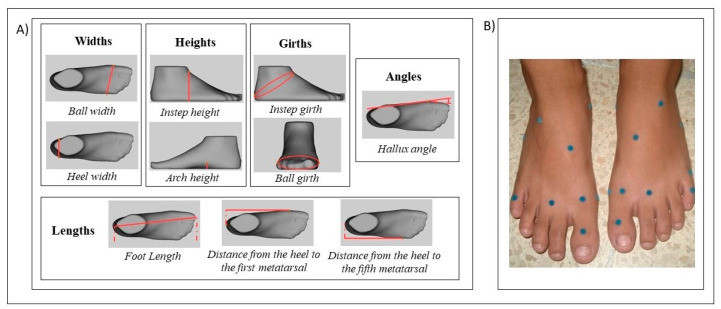
Variables of foot dimensions obtained with 3D scanner; red marks represent the measurement taken after the scanning (**A**). Placement of the 13 skin labels on the foot of participants (**B**).

The variables analyzed (Figure 2) were:

- Foot length (FL): Distance between the most proximal point of the heel and the most distal point of the toes.
- Distance from heel to first metatarsal head (DHMI): distance between the most proximal point of the heel and the medial region of the first metatarsal head.
- Distance from heel to fifth metatarsal head (DHMV): distance between the most proximal point of the heel and the lateral region of the fifth metatarsal head.
- Ball width (BW): distance between the medial region of the first metatarsal head and the lateral region of the fifth metatarsal head.
- Heel width (HW): distance between the most medial point and the most lateral point of the calcaneus.
- Instep height (IH): distance between the highest point of the cuneiform bones and the ground.
- Arch height (AH): height between the most prominent point of the foot arch and the ground.
- Ball girth (BG): The maximum circumference around the metatarsal heads.
- Instep girth (IG): the maximum circumference around the most cranial point of the cuneiform bones.
- Hallux angle (HA): angle formed by the line between the most medial point of the first toe and the most medial point of the first metatarsal head, and another line between the most medial point of the heel and the most medial point of the first metatarsal head.

**Figure 3:**
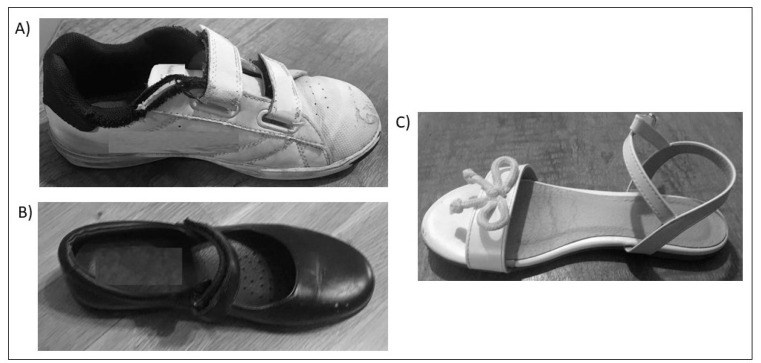
Example of type of footwear most frequently used by participants: sport shoes (**A**); school shoes (**B**); and sandals (**C**).

Lastly, the size and type of footwear used were included. The most typical footwear found was school shoes, sport shoes or sandals (Figure 3). The variable ‘fitting of the foot to footwear’ was calculated by the relationship between the size of the shoe and the length of the foot, according to the French sizing system and scale for the manufacture of footwear [1] and the shoe size used, which corresponded to the size that subjects wore at the time of the analysis.

## 3. Results

No statistically significant differences were found in most variables between left and right foot; therefore, the analyses were performed with mean values of both feet as a representation of the structure of the foot of each subject.

**Figure 4:**
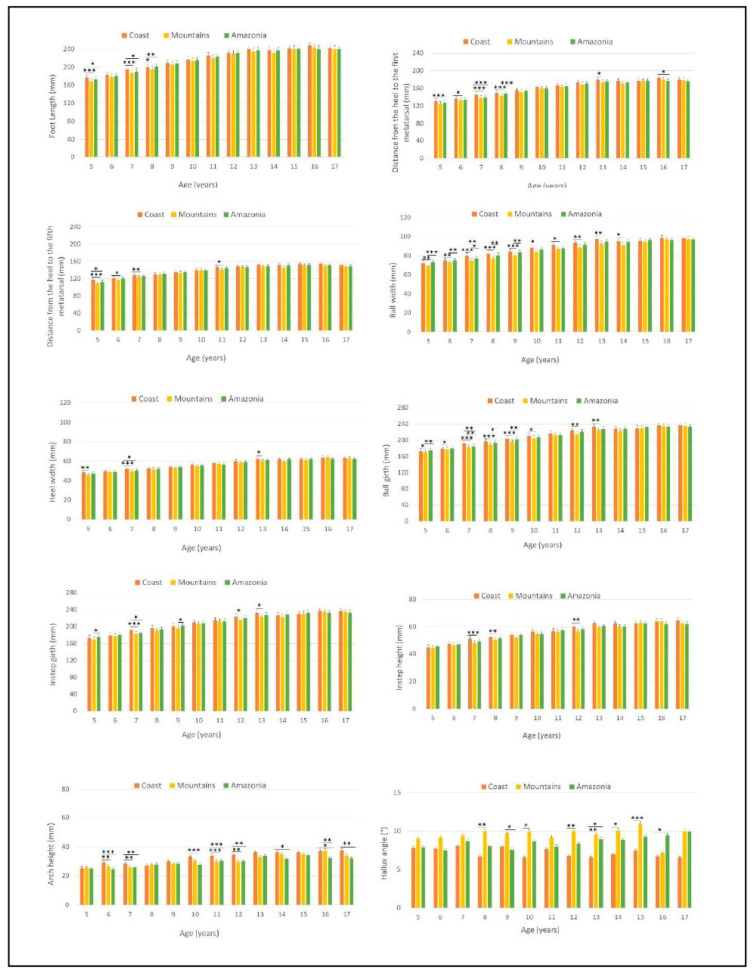
Differences found in variables of foot dimensions according to age between mountain, coast and amazonia groups. Statistical significance: * *p* < 0.05; ** *p* < 0.01; *** *p* < 0.001.

As regards foot dimensions according to geographical location, the three groups showed larger differences at early ages, from 5 to 13. Statistically significant differences were observed in lengths (FL: *p* < 0.001, DHMI: *p* < 0.001 and DHMV: *p* < 0.001), widths (BW: *p* < 0.001) and perimeters (BG: *p* < 0.001 and IG: *p* < 0.001) when comparing the coast and mountain groups. Perimeters (BG: *p* < 0.01) and widths (BW: *p* < 0.001) also showed differences between coastal and amazonian children (Figure 4). From age 10 on, the biggest differences were found in Arch height (AH) when comparing the coast group to the mountain group (*p* < 0.001) and coast to amazonia (*p* < 0.05). The mountain and amazonia groups only showed statistical differences at specific ages for those variables (Figure 4). On the other hand, subjects living in the mountains presented greater values for Hallux angle (HA), obtaining a statistically significant difference compared to the coast group in ages 8 to 17 (*p* < 0.001). When analyzing variables of the foot normalized by foot length, most of the differences are maintained.

Differences based on gender were observed between ages 5 to 9 and 13 to 17, with significantly greater values for boys in lengths (FL: *p* < 0.001, DHMI: *p* < 0.001 and DHMV: *p* < 0.001), widths (BW: *p* < 0.001, HW: *p* < 0.001), perimeters (BG: *p* > 0.001, and IG: *p* < 0.001) and heights (AH: *p* < 0.01 and IH: *p* < 0.01). When analyzing variables of the foot normalized by foot length, most of the differences disappear for all age groups, except Hallux angle (*p* > 0.05) where these differences increase significantly.

The most frequent types of footwear used by the subject of the study were as follows:

In the mountain group: 42.2% school shoes, 53.5% sport shoes and 3.3% sandals. In the coast group: 22.4% school shoes, 22.1% sport shoes and 55.5% sandals. The amazonia group used 20.3% school shoes, 40.7% sport shoes and 32.7% sandals.

Finally, differences in the variable ‘fitting of the foot to footwear’ were found at ages 8 to 17 (*p* < 0.01), being the length of the foot significantly greater than the length of the shoe.

## 4. Discussion

The results obtained show, for the first time, morphological differences in the foot of school-aged Ecuadorian boys and girls according to the geographical and environmental characteristics of their place of residence. It should be noted that the main findings were obtained in subjects from the coastal area compared to those from the mountain group (Figure 4), showing greater values in foot dimensions in boys than in girls, especially in growth stages [16]. In warmer areas, the use of open-toed shoes such as sandals is more widespread (55.5%), whilst in colder areas, the most common shoe type was school shoes (42.2%) or sport shoes (53.5%). This is a sign that the use of a type of shoe is conditioned by the climate, and that this shoe type is interfering with foot development in the studied populations, as found by Hollander et al. [17]. The use of sandals is related to longer (*p* < 0.05), wider feet (*p* < 0.01), and greater perimeters (*p* < 0.01) when compared to the values obtained in subjects using closed-toed shoes. These results are in line with other authors who conclude that subjects living in warm climates, Australia and the Philippines, have longer and wider feet [7,18]. However, other studies show greater lengths [6] and widths of the forefoot [8] comparing German to Australian and Brazilian children respectively. Nevertheless, in these studies, the shoe type used by each of the analyzed populations was not indicated. In our study, when comparing the mountain group to the amazonia group, most of these differences disappear (Figure 4). Despite the difference in temperatures in both areas, in the amazonia, rains are frequent throughout the year, which could justify the predominant use of closed-toed shoes in this group (61.1%). These results suggest the influence of climate and of habitual footwear in the dimensions and growth of children’s feet.

The reason behind finding wider feet and feet with greater perimeters in coastal residents could be the use of open-toed shoes or sandals, since they allow the foot to expand during the stance phase in the gait cycle, as opposed to school or sport shoes.

The height of the scaphoid bone presented lower values in the mountain and amazonia groups compared to the coast group (*p* < 0.01) (Figure 4). Greater percentages of flat feet were found in subjects living in urban areas, who used shoes, compared to rural areas, who frequently went barefoot [3,10,17]. Although in our study all the subjects lived in urban areas, this tendency to higher foot arches in the coast group could indicate a better maturation of the foot due to the use of open shoes in warmer areas. This type of footwear provides less stability and more proprioception, forcing the muscles of the feet to make continuous adjustments to maintain their function, which leads to a strengthening of both the active and passive elements of the foot during the gait cycle [3], increasing the height of the arch.

Subjects living in the mountains presented greater angles of the first toe compared to those from the coast in older ages, between 8 and 17 (Figure 4). There is a direct relationship between the fitting of the foot and Hallux Valgus [19,20], being more frequent in women (*p* < 0.05) between 16 and 17 years old, possibly due to fashion trends and the use of pointy-toed shoes [13,21,22]. Up to 95.7% of the subjects living in the mountains used most frequently sport shoes or school shoes. The use of closed shoes, which compress the forefoot area, could cause deformities around the first metatarsal-phalangeal joint, affecting the morphology of the foot.

When normalizing variables with the length of the foot, most of the differences are maintained. This reinforces the hypothesis that environmental factors condition the use of different types of footwear and affects foot proportions, regardless of age.

Lastly, differences are observed in the variable ‘fitting of the foot to footwear’, for most of the group ages (*p* < 0.01) in both boys and girls. Ecuadorian children population used shoes up to one size smaller than needed for the length of their feet, something that could be influenced by the economic level of society. Although this has not been reported, Ecuador is considered one of the poorest countries in Latin America [23]. In most cases, the shoes used were not replaced after a school year, and some even used to belong to older siblings. A bad fitting of the shoe can produce pain and deformities in the foot [5,20,24].

Poor-fitting footwear has been linked to musculoskeletal conditions and pain in the foot of children, affecting their physiological bone development [1,5,25] which could pose a risk to the health of the feet of ecuadorian population. The results of this study could be of great help for companies which design and manufacture children’s footwear, which should take into account the characteristics of the foot morphometry according to the geographical area where their shoes are sold.

## 5. Conclusions

Ecuadorian children living on the coast presented longer and wider feet with greater perimeters and higher foot arches than those in the mountains or amazonia. However, residents of the mountains presented greater angles of the first toe. Ecuadorian children used shoes up to one size smaller than needed for the length of their feet. Both the footwear and the climate, and the fitting of the foot to shoes could be interfering with their physiological development in growth stages.

## Data Availability

The datasets generated during or analyzed during the current study are available from the corresponding author on reasonable request.
